# Hospital Patient Satisfaction With Nursing Care in Saudi Arabia

**DOI:** 10.7759/cureus.67840

**Published:** 2024-08-26

**Authors:** Jazi S Alotaibi

**Affiliations:** 1 Department of Nursing, Majmaah University, Riyadh, SAU

**Keywords:** nursing, saudi arabia, care, satisfaction, patient

## Abstract

Introduction

Patient satisfaction is a critical indicator of health care quality, yet research exploring patient satisfaction with nursing care in Saudi Arabia (SA) remains limited. This study investigated patients' satisfaction with nursing care quality during their hospitalization in SA.

Material and methods

A cross-sectional study was conducted using a convenience sample of 746 patients from five hospitals in five different regions of SA. Data were collected using the Newcastle Satisfaction With Nursing Scale (NSMS), which has been shown to be valid and reliable.

Results

The average overall patient satisfaction with nursing care was 71.16 ± 13.51 out of 95 (75.96%), indicating a high level of satisfaction with the nursing care the patients received. The highest-scoring items of patient satisfaction were the nurses' manners when carrying out their duties, the amount of privacy the nurses provided and their capability to perform their jobs. However, the level of satisfaction regarding the frequency of nurse check-ins, time spent with patients, and support provided to patients' relatives indicated areas for potential improvement. Also, patients with primary education who were divorced or married and those admitted to the gynecology ward were more satisfied with nursing care.

Conclusion

This study’s findings indicate that the patients were highly satisfied with the care their nurses provided, although opportunities for improvement in some areas of nursing care were identified. These findings may encourage nurses to be more attentive in meeting their patients' needs according to best nursing practices. It is recommended that hospital administrators prioritize initiatives to enhance the quality of nursing care, thereby improving overall patient satisfaction.

## Introduction

Providing high-quality health care is an important challenge of health care systems worldwide. According to the World Health Organization, approximately 1.8 billion people worldwide reside in fragile settings that face challenges in delivering quality essential health services [1]. Insufficient health service quality causes 5.7-8.4 million deaths per year in low- and middle-income countries, accounting for up to 15% of all deaths in these settings [1]. Quality nursing care is essential for ensuring patient well-being and must adhere to the highest standards [2]. Nursing staff are considered the cornerstone of health care services because they mostly interact and stay with patients when they receive comprehensive care [2]. Therefore, it is essential to examine the quality of nursing care from the perspective of patients' satisfaction [3]. Evaluating patient satisfaction is crucial for determining health care quality, including nursing care [4]. 

Patient satisfaction with nursing care refers to the degree to which nursing staff meet patients' expectations [4]. Patient satisfaction with nursing care is considered a vital indicator of the quality of health care services and an essential measure for evaluating the performance of health care services in each country's health settings [5-7]. Examining patient satisfaction is an essential approach to addressing patients' needs which can be expected to improve the provision of health care [4]. More specifically, examining patients' satisfaction during their hospital stay is crucial because most patients, when admitted to the hospital, are less able to cope and adapt to negative situations, heightening their emotions and increasing their feelings of anxiety and depression [8]. The need to monitor and assess patient satisfaction is expected to rise due to the increased demand for quality health care [4].

There is a positive relationship between patient satisfaction and patient care outcomes [7]. More satisfied patients are typically more compliant with their health care regimens, which may decrease their length of hospital stay [9]. Increased patient satisfaction with nursing care could enhance patient trust and foster loyalty toward health care providers; for patients, this may also increase their likelihood of returning to or recommending a health care provider [6,10]. Also, patient satisfaction with nursing care is beneficial for managing and preventing increases in health care costs. Suboptimal nursing care may lead to patient dissatisfaction, worsening health conditions, extended hospital stays, and increased health care expenditure [11]. Therefore, nurses and other health care providers should strive to offer consistently high-quality patient services [12].

Several studies have assessed patient satisfaction with nursing care, underscoring its importance in delivering high-quality care. Most of these studies report high levels of patient satisfaction and positive experiences with nursing care [2,13-18]. Locally in Saudi Arabia (SA), few studies examining this topic found that overall patient satisfaction with nursing care was high [18-20]. Conversely, other studies found moderate to low levels of satisfaction [3,21-26].

Few studies have been conducted on patient satisfaction with nursing care in SA, and most were conducted in one region, but they provided valuable insights into patients’ overall satisfaction [18-20,23,27]. This research gap calls for more comprehensive studies across various health care settings to gain a broader understanding of patient satisfaction with nursing care and pinpoint areas for improvement. It is crucial to conduct diverse, culturally, and socio-demographically sensitive studies that evaluate the quality of nursing care from patients' perspectives, thereby enhancing the overall quality of care delivery in SA.

SA has made significant advancements in its health care system, focusing on improving patient satisfaction, experiences, and outcomes. Recognizing the importance of patient satisfaction in SA, health care providers, and policymakers must prioritize increasing patient satisfaction with nursing care because nurses comprise the largest number of health care providers. High levels of patient satisfaction indicate the quality of nursing care and contribute to improved health outcomes and patient loyalty. There is a need for ongoing efforts to address these challenges and continuously improve patient satisfaction with nursing care. Understanding patients' satisfaction with nursing care can assist nurses and hospital managers in identifying the factors contributing to patients' satisfaction, which may impact the overall quality of nursing services [28]. This study aimed to examine the satisfaction level of patients regarding the quality of nursing they received during their hospitalization in SA. Moreover, the study also explored differences in patient satisfaction levels related to the demographic variables of patients, hospital wards, and hospital settings.

## Materials and methods

Study design and setting

A cross-sectional study was used for this study. This study was performed at five hospitals in five regions of SA, including Riyadh, Mecca, Al-Qurayyat, Aseer, and the Eastern region. This study employed a convenience sampling method among all the patients who met the criteria in the selected hospitals. The inclusion criteria were specifically defined: participants had to be at least 18 years old, proficient in reading and writing Arabic, and must have been hospitalized for more than 48 hours. The exclusion criteria were patients under 18 years old, admitted for less than 48 hours, outpatients, day-care patients, not fluent in reading and writing Arabic, and not oriented. 

Ethical approval to perform this study was gained from one of the Ministry of Health's Ethical Review Boards, which is the Institutional Review Board of the Taif Directorate of Health Affairs no (HAP-02-T-067 # 864). The data collection was collected via a Google survey (Google Forms) and at the beginning of the survey, an informed consent form was provided to inform the respondents of the study's nature, including purpose and objectives, risks, advantages, and their rights. If they agreed, they could participate; if not, they could withdraw without consequences. Privacy and voluntary participation were maintained as the researcher did not collect any personally identifiable information that could potentially disclose the identity of the participants. After data collection, the completed survey questionnaires were kept in a locked cabinet in the research office, and only the researcher had access to the questionnaires. Finally, completed questionnaires will be destroyed after three years. 

Sample size calculation

For this study, the sample size was calculated to be 382, considering a margin of error of 5%, confidence interval of 95%, sample proportion of 50%, and 50 thousand population size. To obtain more responses, all patients who met the criteria in the selected hospitals were invited to participate in this study (1,450 patients). Of the 1,450 questionnaires distributed, 746 were completed and returned, resulting in a 51.44% response rate.

Study instrument

The questionnaire used in this study is structured into two parts. The first part consisted of a demographic questionnaire that gathered information about the patient's age, gender, academic qualifications, and marital status. The second part employed the Newcastle Satisfaction With Nursing Scale (NSNS), a validated instrument designed to assess patients' satisfaction with nursing care.

The NSNS was developed by Thomas et al. to assess how patients perceive and feel satisfied with nursing care; it includes 19 items rated on a five-point Likert scale, which ranges from one (not at all satisfied) to five (completely satisfied) [29]. Permission to use the questionnaire was obtained from the questionnaire developer. Previous validations of the NSNS have underscored its robust psychometric properties, exhibiting excellent reliability and construct validity in different settings [3,19,29-35].

In this study, the NSNS demonstrated high reliability, with a Cronbach's alpha of 0.96 and an intra-class correlation of 0.965, reaffirming its effectiveness for accurately measuring patient satisfaction within the study's context.

Data collection

The data collection lasted for three months, from 1 November 2023 to 31 January 2024. After obtaining ethical approval from the designated ethical authorities in the Ministry of Health, the researcher visited the hospitals targeted for data collection. First, the researchers met with the hospital director, and then the nursing director and the researcher explained the nature of the research and presented its objectives to them. After that, a meeting was held with the head nurses, and the researcher explained the nature of the study, its significance, its objectives, and the inclusion criteria required by the participants. The research also demonstrated to the head nurses the ethical considerations, especially the rights of the research participants, such as that participation is optional. They were then given an electronic link that they, in turn, gave to the patients. A link to the questionnaire was then shared with the head nurses and charge nurses via WhatsApp. The researcher's contact information was provided in the online survey to facilitate communication if the participants had questions or needed clarification.

Statistical analysis 

The data were analyzed using SPSS Version 27.0. Descriptive statistics, including frequency, mean, and standard deviation, were employed to characterize the socio-demographic profiles of the participants and assess the level of patient satisfaction. Additionally, t-tests and a one-way ANOVA were utilized to examine whether there were statistically significant differences in patient satisfaction across demographic profiles. 

Some of the the demographic data were non-parametric such as gender, academic qualifications, marital status, and age groups. The responses to items of the NSNS were recorded on a Likert scale with a range of one to five. The Kolmogorov Smirnov test (X2 (746)=0.079, p<0.001) and Shapiro-Wilk test (X2 (746) = 0.956, p<0.001) showed that the total score of the NSNS was not exactly normal. However, manual inspection of the histogram of the frequency distribution plot and the normal Q-Q plot showed that the deviations may be ignored for practical purposes. Moreover, both the inferential statistics that were employed, i.e., independent t-test, and the one-way ANOVA are quite robust and provide reliable results for minor deviations from normality. 

## Results

Participant characteristics

Table 1 shows the number and distribution of the demographic data of the studied patients. It was found that 37.7% of the studied patients were aged from 31 to 40 years, nearly half (52.4%) were male, 47.1% had a bachelor's degree, and 47.6% were single. In addition, it was found that most of the studied patients (34.6%) were admitted to Al-Iman General Hospital, with 45.7% assigned to the surgical ward.

**Table 1:** Number and distribution of demographic data of the studied patients (N = 746)

Item	N	%
Age (years)		
18 - 20	28	3.8
21 - 30	236	31.6
31 - 40	281	37.7
More than 41	201	26.9
Gender		
Male	391	52.4
Female	355	47.6
Academic qualification		
Primary	23	3.1
Secondary	26	3.5
Tertiary	165	22.1
Diploma	122	16.4
Bachelor's degree	351	47.1
Master's degree	52	7.0
PhD degree	7	0.9
Marital status		
Married	279	37.4
Single	355	47.6
Divorced	73	9.8
Widowed	39	5.2
Hospital		
Al-Qurayyat General Hospital	27	3.6
Aseer Central Hospital	66	8.8
King Fahad Hospital Hofuf	211	28.3
Al-Iman General Hospital	258	34.6
King Fahd Hospital in Jeddah	184	24.7
Ward		
Medical	265	35.5
Surgical	341	45.7
Gynecology	87	11.7
Others	53	7.1

Evaluation of nursing care and their stay in a department

Table 2 shows the number and distribution of the patient’s evaluation of their nursing care and stay in the department. The table shows that the majority of the patients (61.5%) evaluated the nursing services received in the department as either very good or excellent

**Table 2:** Distribution of the studied patients regarding their evaluation of nursing care and stay in a department (N = 746)

Item	N	%
Evaluation of nursing care received in a ward		
Bad	37	5.0
Good	154	20.6
Moderate	97	13.0
Very good	245	32.8
Excellent	213	28.6
Evaluation of a stay in a ward		
Bad	46	6.2
Good	185	24.8
Moderate	111	14.9
Very good	229	30.7
Excellent	175	23.5

Patient satisfaction with nursing care

Table 3 shows the mean ± SD of the studied patients regarding patient satisfaction with nursing care. The mean patient satisfaction score with nursing care was 71.16 ± 13.51; when converted to the equivalent of 100, the score was 75.96 out of 100, indicating a high satisfaction rate. 

**Table 3:** Patient satisfaction with nursing care

Item	Mean ± SD
1. The amount of time nurses spent with you	3.576 ± 1.1089
2. How capable nurses were at their job	3.952 ± 0.9750
3. There is always being a nurse around if you need one	3.749 ± 1.1245
4. Number of nurses who know about your care	3.937 ± 1.0389
5. How quickly nurses came when you called for them	3.637 ± 1.0933
6. The way the nurses made you feel at home	3.610 ± 1.1372
7. The amount of information nurses gave to you about your condition and treatment	3.617 ± 1.1259
8. How often nurses checked to see if you were okay	3.562 ± 1.1207
9. Nurses' helpfulness	3.840 ± 1.0644
10. The way nurses explained things to you	3.729 ± 1.0690
11. How nurses helped put your relatives' or friends' minds at rest	3.602 ± 1.1485
12. Nurses' manner of going about their work	3.983 ± 1.0277
13. The type of information nurses gave to you about your condition and treatment	3.714 ± 1.1329
14. Nurses' treatment of you as an individual	3.739 ± 1.1089
15. How nurses listened to your worries and concerns	3.674 ± 1.1351
16. The amount of freedom you were given on the ward	3.678 ± 1.1249
17. How willing nurses were to respond to your request	3.761 ± 1.1110
18. The amount of privacy nurses gave you	3.956 ± 1.0890
19. Nurses awareness of your needs on average	3.853 ± 1.0941
Overall mean	71.1689 (out of 95) ± 13.50629

The three items with the highest mean values were ''nurse's manner in going about their work'' (3.98 ± 1.03), "the amount of privacy nurses gave you" (3.956 ± 1.0890), and "how capable nurses were at their job" (3.95 ± 0.98). However, the three items with the lowest mean values were "how often nurses checked to see if you were okay" (3.562 ± 1.1207), ''the amount of time nurses spent with you'' (3.576 ± 1.1089), and "how nurses helped put your relatives' or friends' minds at rest" (3.602 ± 1.1485). 

Relationship between patient characteristics and patient satisfaction with nursing care

Table 4 shows the relationship between the patient's degree of satisfaction (overall mean of the patient satisfaction questionnaire) and study variables. It was found that there was a statistically significant association between the patient's degree of satisfaction and academic qualification (F = 6.618, p < 0.001). Those with primary school had a higher satisfaction score (78.61 ± 12.64), while those with a PhD degree had a lower satisfaction score (60.71 ± 23.72). It was found that there was a statistically significant association between the patient's satisfaction with nursing care and marital status (F = 2.768, p = 0.041). Divorcees had the highest satisfaction score (73.48 ± 13.92), while singles had the lowest satisfaction score (69.81 ± 13.68). It was found that there was a statistically significant association between a patient's degree of satisfaction and a medical ward (F = 3.018, p = 0.029). Patients in the gynecology ward had the highest satisfaction score (73.02 ± 13.20), while patients in the surgical ward had the lowest satisfaction score (69.70 ± 13.77).

**Table 4:** Relationship between patients' degree of satisfaction with study variables For two groups, T-test; for three or more groups, one-way analysis of variance (ANOVA).

Item		Mean ± SD	Test of significance	P
Gender	Male	70.7570 ± 13.38521	T = 0.874	0.690
	Female	71.6225 ± 13.64288		
Age	From 18 to 20 Y	75.9286 ± 14.69172	F = 1.770	0.133
	From 21 to 30 Y	71.7034 ± 12.61597		
	From 31 to 40 Y	71.4413 ± 13.58772		
	More than 41 Y	69.4900 ± 14.12258		
Academic qualification	Primary 1	78.6087 ± 12.64098	F = 6.618	<0.001
	Secondary 2	72.2692 ± 12.28514		
	Tertiary	72.8485 ± 13.01528		
	Diploma	72.8607 ± 12.62119		
	Bachelor's degree	70.7265 ± 12.72879		
	Master's degree	62.4231 ± 16.88641		
	PhD degree	60.7143 ± 23.72561		
Marital status	Married	72.4122 ± 12.80114	F = 2.768	0.041
	Single	69.8113 ± 13.67628		
	Divorced	73.4795 ± 13.91852		
	Widowed	70.3077 ± 15.11561		
Hospitals	Al-Qurayyat General Hospital	71.0303 ± 13.84304	F = 1.325	0.251
	Aseer Central Hospital	71.6256 ± 12.50513		
	King Fahad Hospital Hofuf	71.4070 ± 13.58677		
	Al-Iman General Hospital	69.6250 ± 14.07574		
	King Fahd Hospital in Jeddah	76.3846 ± 15.10252		
Ward	Medical	72.6264 ± 12.83579	F = 3.018	0.029
	Surgical	69.7009 ± 13.77311		
	Gynecology	73.0230 ± 13.20410		
	Other	70.2830 ± 14.72619		

## Discussion

This study aimed to examine patient satisfaction with nursing care provided during hospital stays in SA. An analysis of the survey responses revealed that most patients expressed satisfaction with the nursing care they received. Overall, patient satisfaction with nursing care was high, with a total mean of 71.16 ± 13.51, which is equivalent to a 75.96% satisfaction score. This result highlights the positive outcomes of nursing care delivery in SA. The significance of this finding lies in the fact that patient satisfaction is a pivotal metric for gauging the quality of hospital care [36]. This metric is not only a reflection of the health care standards in SA but also a critical benchmark for continuous improvement in nursing practices.

The study's finding aligns with similar research conducted in other regions, including Oman, Turkey, Spain, Iran, Kenya, and India, which also reported high levels of patient satisfaction [2,13-17,37]. Also, the results of the present study align with those of previous studies in SA, which reported a high level of satisfaction with nursing care among patients in SA [18-20,27]. However, our results also contrast with those of international and local studies that found low or moderate patient satisfaction with nursing care, as in other studies [3,21,22,24,25,31]. Locally, some studies performed in SA were inconsistent, such as the study done by Al Qahtani and Al Dahi who reported low-to-medium patient satisfaction with nursing care [23]. Such comparisons are invaluable as they provide a broader context for understanding regional differences and similarities in patient perceptions of nursing care. 

A possible explanation for our findings may be related to nurses' preparedness, mainly from the good working environment at the Ministry of Health hospitals, including recruitment standards, financial benefits, and retaining a skilled and dedicated nursing workforce, which contributed to high patient satisfaction, enhancing the safety and quality of hospital care [19]. Well-trained and highly educated nurses will likely provide higher-quality care and improve patient satisfaction through effective management, clear communication, and empathy. Another explanation is that high patient satisfaction ratings with nursing care might stem from patients’ lack of understanding of nursing care, leading them to subliminal care they reserve [38].

In a deeper examination of the factors influencing satisfaction, our findings indicated that the highest levels of satisfaction were directly linked to the nurses' demeanor and proficiency in performing their duties. Patients highly valued the professionalism and skills with which nurses executed their responsibilities, suggesting that the competencies and interpersonal skills of nursing staff are crucial determinants of patient satisfaction. This observation is consistent with findings from previous studies, which highlighted the impact of nursing competence on patient satisfaction outcomes [16,32,33].

Also, the amount of privacy nurses provided to the patient was one of the most important factors influencing patient satisfaction. This result is consistent with the study done by Akin and Erdogan, which found that the patient's privacy is one of the least items that patients were satisfied with [39]. Respecting patients' privacy suggests that they value feeling respected and secure during their care experience, which builds trust and comfort and enhances their overall perception of the quality of care received. However, the result of this study was inconsistent with some previous studies, which found that the amount of privacy for the patient was the source of low patient satisfaction with nursing care [3,4,35].

The least satisfied items that patients had in this study included how often nurses checked to see if they were okay, how much time nurses spent with them, and how nurses helped their relatives or friends. These results include those of previous studies [3,19,32,40]. The possible explanation for this result is the nursing shortage, which leads to a low nurse-to-patient ratio and performing non-nursing activities, which in turn leads to less time spent with each patient, possibly leading to suboptimal care [32]. Providing sufficient time to communicate with patients, listen to them, and provide the requisite information is essential for patient satisfaction [2,32]. Consequently, receiving sufficient information significantly impacts patients' confidence and satisfaction, making it the key factor in encouraging them to participate in their treatment process [2]. Additionally, informing patients and their families about patients' conditions and treatment processes is crucial in alleviating their fear of the unknown [2].

These insights are immensely beneficial for hospital administrators in SA, providing a clear direction for future improvements in patient care services. By addressing these identified areas, administrators can foster an environment that not only boosts patient satisfaction but also enhances the overall quality of health care delivery. This strategic focus on professional development and evidence-based practice is essential for advancing health care outcomes and maintaining high standards of nursing care.

The study uncovered notable differences in patient satisfaction with nursing care when examining demographic characteristics, such as educational level, marital status, and the unit in which the patient was admitted. Interestingly, patients with primary school education reported higher levels of satisfaction with nursing care compared to those holding higher qualifications, such as a master's degree or PhD. This observation aligns with the findings of previous studies, which also identified that patients with low educational status have more patient satisfaction [2,3,16,18,20]. One explanation for this result is that patients with higher education levels may have higher expectations, a more critical view of the quality of care they receive, and may be more aware of their rights, leading them to be less satisfied with nursing care. However, it should be noted that some studies were not consistent with some previous studies that found a positive relationship between patient qualification levels and their satisfaction with nursing care [22,23]. Also, these findings contrast with those of who reported no significant differences in patient satisfaction based on their educational level [19]. 

In terms of marital status, this study observed a distinct pattern in satisfaction scores: divorcees and married participants exhibited the highest levels of satisfaction, whereas single individuals reported the lowest. To some extent, the results of this study are consistent with the studies that found that married patients were more satisfied with nursing care than other patients [2,22,27]. This finding corroborates the results of Alsaqri, suggesting that marital status can influence patient experiences and satisfaction with nursing care [27]. These variations might be attributed to differing support systems, emotional states, or health care needs among the different groups.

This study highlights significant disparities in patient satisfaction across different hospital wards, revealing that satisfaction levels can be significantly influenced by the specific ward in which care is received. Patients in the gynecology and medical wards reported the highest levels of satisfaction, while those in the surgical ward were notably less satisfied. Patients in surgical wards often experience more stress and discomfort, potentially leading to lower satisfaction scores. These findings are consistent with the results of previous research, which indicated that patients in surgical wards generally report lower satisfaction with nursing care compared to those in gynecology [22,32].

However, the results of this study disagree with some previous studies, which found that patients in surgical units were more satisfied with nursing care than patients in obstetrics units [22,39,41]. This could be related to the different nature of care and interactions in gynecology wards, which might often involve less invasive treatments and potentially more positive outcomes compared to more intensive care settings. 

This study has several limitations. First, it was conducted in the hospitals in different regions in SA to enhance the robustness and wider applicability of these findings; future research should extend this study's model to different settings such as primary health care and specialist hospitals. Second, this study only considered patients' perspectives on their satisfaction with nursing care quality. Future studies should include nurses' perspectives on their satisfaction with the quality of nursing care. Third, the use of self-reported data may lead to biases, such as social-desirability bias. Future studies should consider using multiple data sources to reduce these biases and provide a more thorough exploration of the construct's studies.

## Conclusions

This present study observed a high level of patient satisfaction with nursing care during hospitalizations in SA. Also, patients treated in gynecological wards were more satisfied with nursing care. Additionally, significant variations in satisfaction were noted based on patients' educational background and marital statuses, with primarily educated, divorced, and married individuals reporting the highest levels of satisfaction. Moreover, patients highlighted the importance of nurses' competency during their stay and their ability to provide high-quality nursing care as key factors contributing to their satisfaction.

Although this study showed that the participants' level of satisfaction with nursing care was 71.16 ± 13.51 (equivalent to a 75.96% score), some recommendations need to be considered to maintain this level of satisfaction or increase the level of patient satisfaction with nursing care. An important recommendation is a need for a supportive and patient-centered environment within hospital settings that includes optimizing nurse-patient ratios and promoting interdisciplinary collaboration, which will help health care institutions better meet the varied needs of their patient populations and ultimately improve overall satisfaction with nursing care in SA.

Another recommendation of this study is to emphasize the need for improved competency in nursing care to improve targeted knowledge acquisition and training to equip nurses with advanced skills and up-to-date knowledge pertinent to their roles to provide better nursing care to their patients. Finally, feedback from patients, such as from regular surveys, is particularly valuable because it highlights a pathway toward more effective and scientifically grounded patient care. By integrating survey findings into everyday clinical procedures, nurses can ensure that their practices are aligned with the latest and most effective standards in health care. 

## References

[1] (2023). World Health Organization. Quality health services: A key fact. https://www.who.int/news-room/fact-sheets/detail/quality-health-services.

[2] Karaca A, Durna Z (2019). Patient satisfaction with the quality of nursing care. Nurs Open.

[3] Sharew NT, Bizuneh HT, Assefa HK, Habtewold TD (2018). Investigating admitted patients' satisfaction with nursing care at Debre Berhan Referral Hospital in Ethiopia: a cross-sectional study. BMJ Open.

[4] Alhussin EM, Mohamed SA, Hassan AA, Al-Qudimat AR, Doaib AM, Alhawsawy ED (2024). Patients’ satisfaction with the quality of nursing care: a cross-section study. Int J Afr Nurs Sci.

[5] Gishu T, Weldetsadik AY, Tekleab AM (2019). Patients' perception of quality of nursing care; a tertiary center experience from Ethiopia. BMC Nurs.

[6] Liu S, Li G, Liu N, Hongwei W (2021). The impact of patient satisfaction on patient loyalty with the mediating effect of patient trust. Inquiry.

[7] Mulugeta H, Wagnew F, Dessie G, Biresaw H, Habtewold TD (2019). Patient satisfaction with nursing care in Ethiopia: a systematic review and meta-analysis. BMC Nurs.

[8] Alzahrani N (2021). The effect of hospitalization on patients' emotional and psychological well-being among adult patients: an integrative review. Appl Nurs Res.

[9] Aiken LH, Sloane DM, Ball J, Bruyneel L, Rafferty AM, Griffiths P (2018). Patient satisfaction with hospital care and nurses in England: an observational study. BMJ Open.

[10] Hepsiba RP, Bhattacharjee TA (2021). A comparative study to assess the level of patient satisfaction on quality of nursing care among parturients admitted in government and private hospitals at Lucknow. Ann Rom Soc Cell Biol.

[11] Brooks Carthon JM, Hatfield L, Brom H, Houton M, Kelly-Hellyer E, Schlak A, Aiken LH (2021). System-level improvements in work environments lead to lower nurse burnout and higher patient satisfaction. J Nurs Care Qual.

[12] Fatima T, Malik SA, Shabbir A (2018). Hospital healthcare service quality, patient satisfaction and loyalty: an investigation in context of private healthcare systems. Int J Qual Reliab Manag.

[13] Sharma A, Kasar PK, Sharma R (2014). Patient satisfaction about hospital services: a study from the outpatient department of tertiary care hospital, Jabalpur, Madhya Pradesh, India. Indian J Community Med.

[14] Romero-García M, Delgado-Hito P, de la Cueva-Ariza L (2019). Level of satisfaction of critical care patients regarding the nursing care received: correlation with sociodemographic and clinical variables. Aust Crit Care.

[15] Ndambuki J (2013). The level of patients’ satisfaction and perception on quality of nursing services in the Renal unit, Kenyatta National Hospital Nairobi, Kenya. Open J Nurs.

[16] Farmahini Farahani M, Shamsikhani S, Sajadi Hezaveh M (2014). Patient satisfaction with nursing and medical care in hospitals affiliated to Arak University of Medical Sciences in 2009. Nurs Midwifery Stud.

[17] Albashayreh A, Al-Rawajfah OM, Al-Awaisi H, Karkada S, Al Sabei SD (2019). Psychometric properties of an Arabic version of the patient satisfaction with nursing care quality questionnaire. J Nurs Res.

[18] Alhowaymel F, Abaoud A, Alhuwaimel A, Alenezi A, Alsayed N (2022). COVID-19 patients’ satisfaction levels with nursing care: a cross-sectional study. SAGE Open Nurs.

[19] Alasad J, Abu Tabar N, AbuRuz ME (2015). Patient satisfaction with nursing care: measuring outcomes in an international setting. J Nurs Adm.

[20] Alharbi HF, Alzahrani NS, Almarwani AM, Asiri SA, Alhowaymel FM (2023). Patients' satisfaction with nursing care quality and associated factors: a cross-section study. Nurs Open.

[21] Elayan RM, Ahmad MM (2018). A new approach in exploring satisfaction with nursing care by nurses themselves. J Clin Nurs.

[22] Kasa AS, Gedamu H (2019). Predictors of adult patient satisfaction with nursing care in public hospitals of Amhara region, Northwest Ethiopia. BMC Health Serv Res.

[23] Al Qahtani M, Al Dahi SK (2015). Satisfaction with nursing care from the Inpatients' perspective in Prince Salman Armed Forced Hospital Tabuk, Saudi Arabia. Middle East J Fam Med.

[24] Hajy MA, Ahmed KM, Ahmed NS, Ahmed HM (2024). Patient satisfaction with nursing care based on Newcastle satisfaction with nursing scale in Erbil/Iraq. Continuity.

[25] Goh ML, Ang EN, Chan YH, He HG, Vehviläinen-Julkunen K (2016). A descriptive quantitative study on multi-ethnic patient satisfaction with nursing care measured by the Revised Humane Caring Scale. Appl Nurs Res.

[26] Khaleel MA, Al-Hussein RA (2015). Assessment of patients satisfaction regarding nursing care provided at general hospitals in Al-Najaf city. Kufa J Nurs Sci.

[27] Alsaqri Alsaqri, S S (2016). Patient satisfaction with quality of nursing care at governmental hospitals, Ha’il City, Saudi Arabia. J Bio Agri Healthc.

[28] Lotfi M, Zamanzadeh V, Valizadeh L, Khajehgoodari M (2019). Assessment of nurse-patient communication and patient satisfaction from nursing care. Nurs Open.

[29] Thomas LH, McColl E, Priest J, Bond S, Boys RJ (1996). Newcastle satisfaction with nursing scales: an instrument for quality assessments of nursing care. Qual Health Care.

[30] Piredda M, Vellone E, Piras G (2015). Psychometric evaluation of the Newcastle Satisfaction with Nursing Scales. J Nurs Care Qual.

[31] Wudu MA (2021). Predictors of adult patient satisfaction with inpatient nursing care in public hospitals of Eastern Amhara Region, northeastern Ethiopia, 2020. Patient Prefer Adherence.

[32] Alhusban MA, Abualrub RF (2009). Patient satisfaction with nursing care in Jordan. J Nurs Manag.

[33] Alasad JA, Ahmad MM (2003). Patients’ satisfaction with nursing care in Jordan. Int J Health Care Qual Assur.

[34] Peterson WE, Charles C, DiCenso A, Sword W (2005). The Newcastle Satisfaction with Nursing Scales: a valid measure of maternal satisfaction with inpatient postpartum nursing care. J Adv Nurs.

[35] Legesse MT, Walle A (2016). Adult patient satisfaction with in-patient nursing care in a referral and teaching Hospital in Southern Nations Nationalities and Peoples’ Region (SNNPR), Ethiopia. J Nurs Care.

[36] Ipek Coban G, Kasikci M (2010). Reliability and validity of the scale of patient perception of hospital experience with nursing care in a Turkish population. J Clin Nurs.

[37] Shinde M, Kapurkar K (2014). Patient’s satisfaction with nursing care provided in selected areas of tertiary care hospital. Int J Sci Res.

[38] Burhans LM (2008). What is good nursing care? The lived meaning of quality nursing care for practicing nurses (Doctor of Philosophy). East Carolina University.

[39] Akin S, Erdogan S (2007). The Turkish version of the Newcastle Satisfaction with Nursing Care Scale used on medical and surgical patients. J Clin Nurs.

[40] Ahmed T, Assefa N, Demisie A, Kenay A (2014). Levels of adult patients’ satisfaction with nursing care in selected public hospitals in Ethiopia. Int J Health Sci (Qassim).

[41] Geckil E, Dundar O, Sahin T (2008). Evaluation of patients’ satisfaction levels from nursing care at the centre of the city Adıyaman. Hacet Üniv J Nurs.

